# Virulence of Japanese Encephalitis Virus Genotypes I and III, Taiwan

**DOI:** 10.3201/eid2311.161443

**Published:** 2017-11

**Authors:** Yi-Chin Fan, Jen-Wei Lin, Shu-Ying Liao, Jo-Mei Chen, Yi-Ying Chen, Hsien-Chung Chiu, Chen-Chang Shih, Chi-Ming Chen, Ruey-Yi Chang, Chwan-Chuen King, Wei-June Chen, Yi-Ting Ko, Chao-Chin Chang, Shyan-Song Chiou

**Affiliations:** National Chung Hsing University, Taichung, Taiwan (Y.-C. Fan, J.-W. Lin, S.-Y. Liao, J.-M. Chen, Y.-Y. Chen, C.-M. Chen, Y.-T. Ko, C.-C. Chang, S.-S. Chiou);; National Defense Medical Center, Taipei, Taiwan (H.-C. Chiu); Tri-Service General Hospital (H.-C. Chiu);; Taiwan Mennonite Christian Hospital, Hualien, Taiwan (C.-C. Shih);; Tungs’ Taichung MetroHarbor Hospital, Taichung (C.-M. Chen);; National Dong Hwa University, Hualien (R.-Y. Chang);; National Taiwan University, Taipei (C.-C. King);; Chang Gung University, Taoyuan, Taiwan (W.-J. Chen)

**Keywords:** Japanese encephalitis virus, genotype, GI JEV, GIII JEV, flavivirus, mosquitoborne, vector-borne infections, virulence, asymptomatic ratio, Asia, Western Pacific, Taiwan, viruses, meningitis/encephalitis

## Abstract

The virulence of genotype I (GI) Japanese encephalitis virus (JEV) is under debate. We investigated differences in the virulence of GI and GIII JEV by calculating asymptomatic ratios based on serologic studies during GI- and GIII-JEV endemic periods. The results suggested equal virulence of GI and GIII JEV among humans.

Japanese encephalitis virus (JEV), a mosquitoborne flavivirus, causes Japanese encephalitis (JE). This virus has been reported in Southeast Asia and Western Pacific regions since it emerged during the 1870s in Japan (*1*). JEVs are divided into 5 genotypes on the basis of envelope structural protein genes for phylogenetic reconstruction. JEV genotype III (GIII) has been the most widely distributed in the temperate zone and is most frequently associated with JEV outbreaks in Asia. JEV genotype I (GI) originated in Indonesia and circulated in Thailand and Cambodia during the 1970s (*1*). Dominance of GIII was replaced by GI during 1992–2001 in Japan, Korea, Thailand, and Vietnam (*1*).

In Japan, the confirmed case incidence of JEV suddenly decreased from 20–50 cases each year during 1980–1990 to <10 cases after 1992 (*2*). This decrease may be related to implementation of JEV vaccine in Japan but also to less virulent GI JE viruses circulating there (*3*–*6*). A similar decrease was not seen in the other countries where genotype replacement had also occurred in recent years (*7*). In Taiwan, JEV GI was first detected in 2008 and became the island-wide dominant circulating genotype within a year (*8*,*9*), which provided an excellent opportunity to study the transmission dynamics and pathogenicity of these 2 JEV genotypes.

A mouse model showed that the pathogenic potential is similar among different JEV genotypes (*10*). However, the pathogenic difference between GI and GIII virus infections among humans remains unclear. Endy et al. reported that the proportion of asymptomatic infected persons among total infected persons (asymptomatic ratio) is an excellent indicator for estimating virulence or pathogenicity of dengue virus infections among humans (*11*). We used the asymptomatic ratio method for a study to determine if GI JEV is associated with lower virulence than GIII JEV among humans in Taiwan.

## The Study

JEVs were identified in 6 locations in Taiwan during 1994–2012 (Technical Appendix Figure, panel A). GIII viruses were the only known circulating JEVs in Taiwan before 2009 (Technical Appendix Figure, panel B). A genotype shift was complete by 2009, and since then, all JEV isolates in Taiwan have evolved from GI viruses (*8*). To investigate differences in the virulence of GIII and GI viruses in human infections, we conducted a subcohort and cross-sectional combined study to determine the JEV asymptomatic infection ratio. We used serum panels collected during the GIII JEV endemic period (1994–2000 [*12**,**13*]) and during the GI JEV endemic pe-riod (2010–2012).

The institutional review boards of the Mennonite Christian Hospital and the Tungs’ Taichung Metroharbor Hospital reviewed and approved clinical protocols for the serum sample collection. Approximately 10% of total specimens were paired serum samples. We used the plaque reduction neutralization test (PRNT) and IgM antibody-capture ELISA (MAC-ELISA) to determine the infection status of each serum specimen (*14*). We tested serum panels collected before and after 2009 by using MAC-ELISA and PRNT and used viral antigens and viruses derived from the GIII-T1P1 and GI-YL2009–4 strains, respectively.

We further tested all neutralizing and IgM antibody-positive specimens by using GI YL2009–4, GIII T1P1, and dengue virus 2 (DENV-2) viral antigens to determine the genotype-specific infection status and to exclude false-positive results possibly caused by DENV infection. We used SAS version 9.4 (SAS Institute Inc., Cary, NC, USA) for statistical analyses.

We collected 5,557 specimens from 4,617 participants (Table 1). The average participant age by year ranged from 40.9 to 52.0 years (p<0.05); overall, 49.6% of participants were male and 50.4% female (p>0.05). The proportion of persons receiving JEV vaccinations increased over time from 33% to 49% (p<0.05).

**Table 1:** Descriptive characteristics of populations for study of virulence of JEV genotypes I and III, Taiwan*

Characteristic	Region								p value†
	Changhua	Taipei	Pingtung	Miaoli	Taichung	Hualien	Taichung	Hualien	
Year	1994	1995	1999	2000	2010	2010	2012	2012	ND
Circulating JEV	GIII	GIII	GIII	GIII	GI	GI	GI	GI	ND
No. participants	795	886	571	274	510	754	527	300	ND
No. (%) vaccinated	36	41	41	33	48	48	49	46	<0.05
Average age, y‡	42.8	40.9	47.1	51.3	49.6	48.1	52.0	49.2	<0.05
Male sex, %	45	52	47	54	50	48	52	49	>0.05

*JEV, Japanese encephalitis virus; ND, no data. †Analyzed by using 1-way analysis of variance. ‡Range 40.9–52.0 y.

The positivity rate for neutralizing antibody, elicited by natural infection or immunization in the past, differed significantly, ranging from 58% to 75% among the 8 serum panels (p<0.05) (Table 2). A JEV infection that had been acquired recently was indicated by the presence of IgM among the seronegative population, seroconversion of the neutralizing antibody, and a ≥4-fold increase in the neutralizing antibody titer among the seropositive population. For the 8 serum panels, the IgM positivity rate ranged from 0.90% to 4.91% (p<0.05), and the proportion of seroconversion and a ≥4-fold increase in the neutralizing antibody titer ranged from 0% to 1.37% (p>0.05). The incidence rate of JEV infection ranged from 1.83% to 5.49% (p<0.05) in the 8 serum panels.

**Table 2:** Estimated incidence of infection and asymptomatic ratios of JEV genotypes I and III among study populations, Taiwan*

	Region								
Characteristic	Changhua	Taipei	Pingtung	Miaoli	Taichung	Hualien	Taichung	Hualien	p value†
Year	1994	1995	1999	2000	2010	2010	2012	2012	ND
No. specimens (incidence of infection)	966 (171)	974 (88)	638 (67)	411 (137)	656 (146)	905 (151)	632 (105)	375 (75)	ND
NT positive, %	62	69	60	58	65	75	72	66	<0.05
IgM positive, % (A)	4.91	0.90	1.93	1.10	0.98	2.25	3.23	3.67	<0.05
Seroconversion + ≥4-fold increase in NT titer, %‡ (B)	0.58	1.13	0	0.73	1.37	1.32	0.95	1.33	>0.05
Incidence of infection, % (C = A + B)	5.49	2.03	1.93	1.83	2.35	3.57	4.18	5.00	<0.05
Population§ (D)	483,766	2,643,221	909,778	559,703	598,186	339,659	593,780	337,382	ND
Predicted no. infections (E = C × D)	26,559	53,657	17,559	10,243	14,057	12,126	24,820	16,869	ND
Confirmed cases (F)¶	4	3	2	1	2	2	2	2	ND
Asymptomatic ratio (G = F/E)	1/6,640	1/17,886	1/8,779	1/10,243	1/7,029	1/6,063	1/12,410	1/8,435	>0.05
Asymptomatic ratio, 95% CI	1/5,102–1/8,000	1/15,408–1/21,322	1/8,000–1/9,709	1/8,065–1/13,947	1/6,329–1/7,874	1/5,348–1/6,993	1/10,846–1/14,493	1/7,299–1/10,010	ND

*Serum samples were collected after the Japanese encephalitis season (May–September); multiple serum samples were collected from some participants. JEV, Japanese encephalitis virus; ND, no data; NT, neutralizing antibody.  †Analyzed by using one-way analysis of variance. ‡Among the IgM-negative population, JEV infection was estimated by the observation of an increase in the NT titer, including seroconversion and ≥4-fold increase in the NT titer among subjects with multiple serum samples. §In Taichung, only residents of the districts around the serum-collecting hospital were included. ¶According to the official report of the Taiwan Centers for Disease Control.

We calculated the asymptomatic ratio by dividing the number of confirmed JE cases by the number of JEV infection cases. The number of confirmed JE cases (Table 2) was obtained from the CDC National Infectious Disease Statistics System, Taiwan (https://nidss.cdc.gov.tw/en/Default.aspx), which reported 1–4 cases/year in the regions from which the 8 serum panels were collected (Table 2). The number of JEV infection cases, calculated by multiplying the total population by the incidence of JEV infection, ranged from 10,243 to 53,657 in the 8 serum panels. The asymptomatic ratios (95% CI) for GIII virus infection were 1:6,640 (1:5,102–1:8,000) for Changhua County in 1994; 1:17,886 (1:15,408–1:21,322) for Taipei City in 1995; 1:8,779 (1:8,000–1:9,709) for Pingtung County in 1999; and 1:10,243 (1:8,065–1:13,947), for Miaoli County in 2000. The asymptomatic ratios for GI virus infection were 1:7,029 (1:6,329–1:7,874) for Taichung City and 1:6,063 (1:5,348–1:6,993) for Hualien County in 2010; ratios were 1:12,410 (1:10,846–1:14,493) for Taichung City and 1:8,435 (1:7,229–1:10,010) for Hualien County in 2012.

We applied the log-linear Poisson regression model to adjust the asymptomatic ratio by the number of patients who had encephalitis and were infected by other pathogens, and also by age, sex, and vaccination status (Technical Appendix Table) (*15*). The results revealed that non–JEV-specific encephalitis symptoms (fever, headache, convulsion, and seizure) (intercept, p<0.05) influenced the calculation of asymptomatic ratios but not age, sex, or vaccination status (p>0.05).

We calculated the overall GI JEV–­ and GIII JEV–specific asymptomatic ratios by using the adjusted asymptomatic ratios of the 8 serum panels collected during the GI JEV– and GIII JEV–endemic periods (Figure). The GI-specific asymptomatic ratio was 1:15,378 (1:6,168–1:24,588) and the GIII-specific ratio was 1:18,842 (1:6,624–1:30,260); these ratios were not significantly different (p>0.05).

**Figure:**
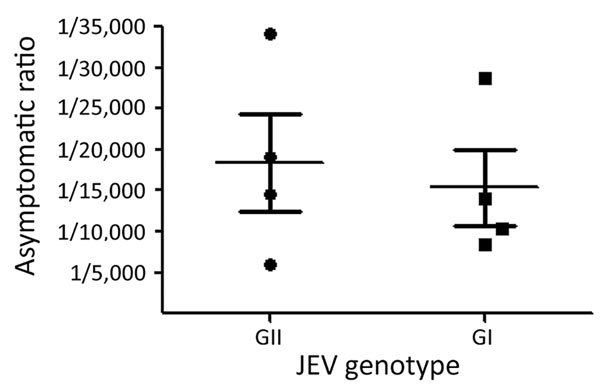
JEV genotype-specific asymptomatic ratios for 8 serum panels collected during the GIII- and GI-endemic periods (1994–2000 and 2010–2012) in Taiwan. The adjusted asymptomatic ratios estimated from genotype-representing populations were included to calculate JEV genotype-specific asymptomatic ratios. Horizontal lines indicate mean; error bars indicate SEM. JEV, Japanese encephalitis virus.

## Conclusions

The possible effects of JEV genotype replacement remain unclear, particularly in terms of disease burden, virulence, vaccine efficacy, and policy decisions. In this study, we combined the IgM seropositivity rate, the proportion of seroconversion, and paired samples displaying a ≥4-fold increase in the neutralizing antibody titer to calculate incidence of the infection (Table 2). The results showed that the incidence of JEV infection fluctuated over years and in different regions in Taiwan. Nevertheless, genotype replacement had no significant effect on this fluctuation (GI:GIII = 3.78 ± 1.25:2.82 ± 3.18; p>0.05).

The dramatic decline observed in the number of clinical JE cases after the genotype replacement from GIII to GI in Japan suggested that GI viruses were less virulent than GIII viruses (*3*–*6*). However, we found that the asymptomatic ratio of GI and GIII JEV infections was similar, indicating equal virulence of GIII and GI JEVs. These results were also supported by the mouse virulence and neurovirulence experiments and disease burden estimation (*10*).

Technical Appendix Prevalence, circulation, and ratios of Japanese encephalitis virus genotypes I and III in cities, regions, and counties of Taiwan.
