# Supplementary material for: Virulence of Japanese Encephalitis Virus Genotypes I and III, Taiwan
Source: Emerg Infect Dis. 2017 Nov;23(11):1883–6. doi: 10.3201/eid2311.161443 (PMC5652437; doi:10.3201/eid2311.161443)
Supplement: Technical Appendix — Prevalence, circulation, and ratios of Japanese encephalitis virus genotypes I and III in cities, regions, and counties of Taiwan. [file 16-1443-Techapp-s1.pdf]

Article DOI: https://doi.org/10.3201/eid2311.161443

Virulence of Japanese Encephalitis Virus Genotypes I and III, Taiwan

Technical Appendix

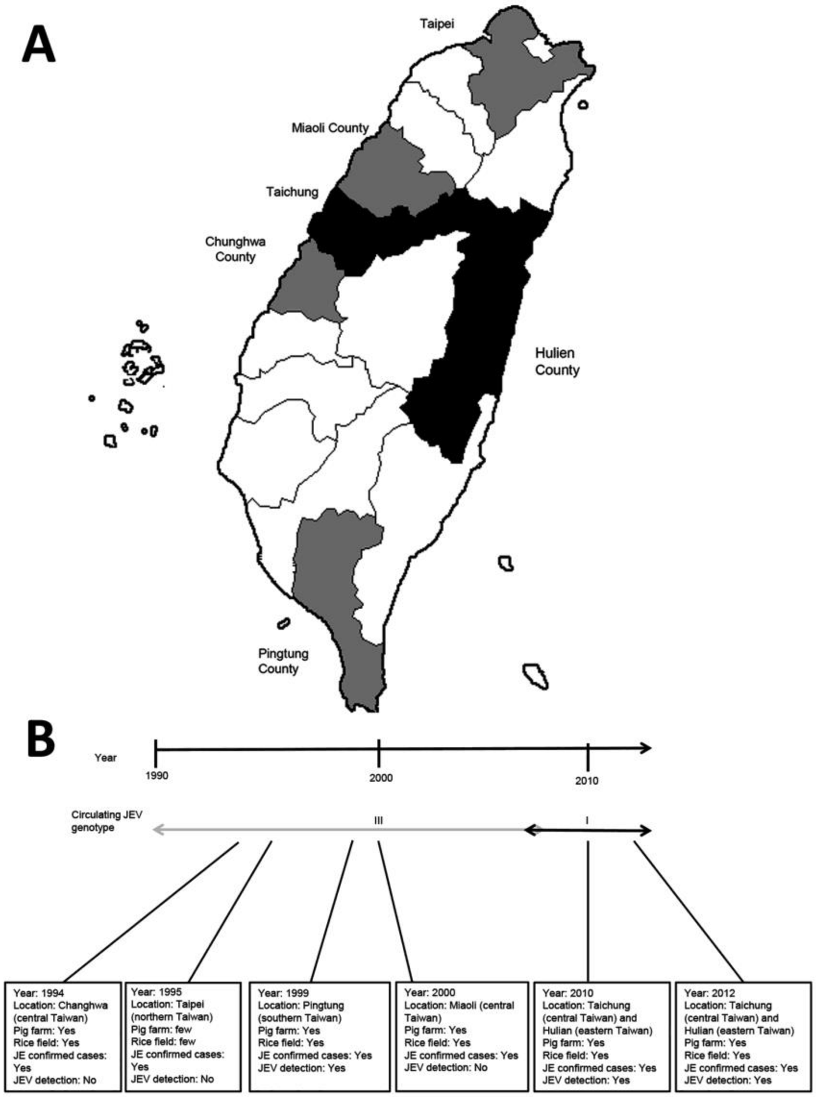

Technical Appendix Figure. A) In 6 counties or cities of Taiwan, prevalence of Japanese encephalitis virus (JEV) genotype I are indicated in gray, and of genotype III in black. B) Circulating Japanese

encephalitis virus (JEV) genotypes (GIII indicated as gray and GI as black lines) in Taiwan. In the boxes, JEV background information on the study areas and populations is provided, including the year of sample collection, location, presence of pig farms, rice fields, confirmed JE cases, and JEV circulation. The ecological conditions, pig farms, and rice fields of these regions were suitable for JEV transmission. Although JEVs were detected in the studied regions according to the previous reports (*1–3*), only half of the regions showed the presence of JEVs during the sampling periods in our study. Confirmed JE cases were identified in these regions in a sampling year according to official reports from the Center for Disease Control (CDC), Taiwan.

Technical Appendix Table. Adjusted asymptomatic ratio using the log-linear Poisson regression model*

	Log-linear Poisson Regression Model			
Variable	Regression Coefficient	Exp (Regression Coefficient)†	Adjusted asymptomatic ratio‡	p-value
Intercept	-0.0000401618	0.999959839	NA	<0.05
Changhua	-0.0001048516	0.999895154	1/5,938	NA
Taipei	-0.0000292712	0.999970729	1/34,164	NA
Pingtung	-0.0000686509	0.999931351	1/14,567	NA
Miaoli	-0.0000523614	0.999947640	1/19,099	NA
Taichung (2010)	-0.0000964151	0.999903590	1/10,372	NA
Hualien (2010)	-0.0001187128	0.999881294	1/8,424	NA
Taichung (2012)	-0.0000347737	0.999965227	1/28,758	NA
Hualien (2012)	-0.0000716391	0.999928363	1/13,959	NA
Age	0.0000015502	1.000001550	NA	>0.05
Gender	0.0000004365	1.000000437	NA	>0.05
Vaccination	-0.0000142803	0.999985720	NA	>0.05

*Both JEV infected and uninfected patients may present symptoms undifferentiated from true JEV to non-JEV infection; in such cases, calculating the asymptomatic ratio becomes difficult. Therefore, we used the log-linear Poisson regression model to adjust the asymptomatic ration by the number of encephalitis patients caused by pathogens other than JEV. The Poisson distribution was applied while considering encephalitis as the rare event, and the log-linear Poisson regression model was modified from the log-linear binomial regression one (*4*). Intercept, non-specific JEV symptoms including fever, headache, convulsion, and seizure; NA, not applicable.
†The exp (regression coefficient) is the adjusted possibility of the asymptomatic infection.
‡The adjusted asymptomatic ratio = 1 / [1–exp (regression coefficient)].

References

1. Chen YY, Fan YC, Tu WC, Chang RY, Shih CC, Lu IH, et al. Japanese encephalitis virus genotype replacement, Taiwan, 2009-2010. Emerg Infect Dis. 2011;17:2354–6. PubMed http://dx.doi.org/10.3201/eid1712.110914

2. Chiou SS, King CC. Japanese encephalitis virus recent infection: detection of IgM antibody. Report of undergraduate student research grant, National Science Council, Taiwan. 1994.

3. Chiou SS, Tsai KH, Huang CG, Liao YK, Chen WJ. High antibody prevalence in an unconventional ecosystem is related to circulation of a low-virulent strain of Japanese encephalitis virus. Vaccine. 2007;25:1437–43. PubMed http://dx.doi.org/10.1016/j.vaccine.2006.10.044

4. Wang TE, Lin CY, King CC, Lee WC. Estimating pathogen-specific asymptomatic ratios. Epidemiology. 2010;21:726–8. PubMed http://dx.doi.org/10.1097/EDE.0b013e3181e94274
